# The Prevalence of Chronic Kidney Disease and Albuminuria in Patients With Type 1 and Type 2 Diabetes Attending a Single Centre

**DOI:** 10.7759/cureus.32248

**Published:** 2022-12-06

**Authors:** MS Majeed, Fahad Ahmed, Mary Teeling

**Affiliations:** 1 Diabetes and Endocrinology, Southeastern Healthcare and Social Trust, Belfast, GBR; 2 Endocrinology and Diabetes, University Hospitals Sussex NHS Foundation Trust, Brighton, GBR; 3 Pharmacology and Therapeutics, Trinity College Dublin, Dublin, IRL

**Keywords:** prevalence, diabetic kidney disease, albuminuria, chronic kidney disease, diabetes

## Abstract

Background

Diabetes is the leading cause of chronic kidney disease worldwide. Diabetic kidney disease is one of the microvascular complications of diabetes and it involves changes in glomerular hemodynamics, interstitial fibrosis, and tubular atrophy. Early detection and management of Diabetic kidney disease (DKD) help to reduce morbidity and mortality. This study aims to assess the prevalence of nephropathy and albuminuria in the diabetic population attending an Irish tertiary care center.

Methods

Retrospective data collection and analysis of patients diagnosed with Type 1 diabetes mellitus (T1DM) and Type 2 diabetes mellitus (T2DM) through the Development and Integration of Accurate Mathematical Operations in Numerical Data-Processing (DIAMOND) database in a single Irish tertiary care center. An audit tool was used to collect patients’ information including gender, age, type of diabetes, serum creatinine, urinary albumin excretion, albumin creatinine ratio (ACR), body mass index, and last available glycated hemoglobin (HbA1c).

Results

Out of 7394 subjects with T2DM, 3139 (42%) were identified with chronic kidney disease (CKD). There were 1866 subjects with positive ACR out of 3139 subjects with CKD in the T2DM cohort. This shows that 25% of subjects have diabetic kidney disease and 17% have CKD of undetermined etiology. In the T1DM cohort with 1166 subjects, 209 (18%) were identified with CKD. Out of these 209 subjects with CKD, 164 (14%) were ACR-positive. The prevalence of CKD and albuminuria were related to age in both T1DM and T2DM populations. Albuminuria showed a linear relationship with age in subjects with no known CKD, which shows that age causally relates to albuminuria independent of type and duration of diabetes.

Conclusion

CKD is more prevalent in patients with T2DM as compared to T1DM, whereas the prevalence of albuminuria is higher in the T1DM population.

## Introduction

Diabetes mellitus (DM) is a chronic metabolic disorder characterized by persistent hyperglycemia due to partial (insulin resistance) or absolute insulin deficiency. The estimated prevalence of diabetes is 4-6% in the United Kingdom and 5.8-12.9% in the United States [1-2]. 

Patients with DM require regular assessment DM-related microvascular (retinopathy, neuropathy, and nephropathy) and macrovascular (coronary artery disease, stroke) complications. As type 2 diabetes (T2DM) is insidious in onset so screening for complications should be started at the time of diagnosis whereas in type 1 diabetes (T1DM) it is advised at five years from the time of diagnosis [3]. Better glycaemic control with attention to associated risk factors is essential in the prevention of diabetic complications.

Kidney disease improving global outcome (KDIGO) defines CKD as abnormalities of kidney structure or function present for > three months, with implications for health [4]. CKD in DM may or may not represent Diabetic kidney disease (DKD). DKD has been classically defined by the presence of proteinuria >0.5 g/24hr urine collection, this stage has also been referred to as overt nephropathy or macro albuminuria [4]. The incipient nephropathy is characterized by microalbuminuria (defined as urinary albumin excretion between 30-300mg/dl). CKD can be attributed to DM if there is macro albuminuria, microalbuminuria with diabetic retinopathy, or microalbuminuria with T1DM. Other causes of CKD should be considered if there is no evidence of DM-related eye disease, rapidly decreasing glomerular filtration rate, especially after the introduction of an angiotensin-converting enzyme inhibitor (ACEI) or angiotensin receptor blocker (ARB), increasing proteinuria, refractory hypertension, presence of active urinary sediments or signs and symptoms of other systemic illness.

DKD is one of the microvascular complications of DM and is pathologically characterized by mesangial expansion, glomerular basement membrane thickening, and glomerular sclerosis. Albuminuria and progressive decrease in eGFR are major clinical manifestations of DKD, less frequently haematuria can also be observed. Microalbuminuria is the marker of endothelial dysfunction and an independent risk factor for cardiovascular disease however there is accumulating evidence that the risk of developing DKD and cardiovascular disease starts at normal urinary albumin excretion [5]. DKD is associated with increased morbidity and mortality especially cardiovascular disease. Poor diabetic control, high blood pressure, and genetic predisposition are the main risk factors for the development of DKD. Raised serum lipids, smoking, and dietary proteins also play a role as additional risk factors [6].

European Diabetes prospective complications study group (EURODIAB) has shown a cumulative incidence of microalbuminuria in T1DM was 12.6% at 7.3 years and nearly 33% at 18 years follow-up study in Denmark [5,7]. In the U.K. Prospective Diabetes Study (UKPDS) [8] over a median of 15 years from diagnosis of type 2 diabetes, nearly 40% of patients developed albuminuria, and nearly 30% developed renal impairment. incidence of microalbuminuria in T2DM was 2%. The estimated prevalence of incipient and overt diabetic nephropathy is 28.1% and 6.1% in the US, 19% and 6.8% in the UK, and 27% and 14% in European populations, respectively [9-10]. 

There has been no previous study looking at the prevalence of CKD in the Irish DM population. Gemma et al. looked into the prevalence of diminished kidney function in middle and older age groups in the Irish population independent of DM. The prevalence of CKD was estimated to be 11.6% (95%CI 9.0%-14.2%) [10]. Our study aimed to assess the prevalence of diabetic nephropathy and albuminuria in a population with DM attending a tertiary care center. Furthermore, we evaluated the prevalence of T1DM and T2DM nephropathy and albuminuria in different age categories.

## Materials and methods

Study sample

The study sample was taken from the Development and Integration of Accurate Mathematical Operations in Numerical Data-Processing (DIAMOND) database, which includes all patients with DM attending tertiary care center, Adelaide, Meath and National Children Hospital (AMNCH) Dublin, Republic of Ireland. The study was approved by Saint James Hospital and Adelaide, Meath and National Children Hospital (SJH/AMNCH) Research Ethics committee. DIAMOND database stores basic information about patients with diabetes including age, gender, Blood pressure, Body mass index, type of DM, and their basic investigations including HbA1c.

Inclusion and exclusion criteria

Inclusion Criteria

**Figure 1:**
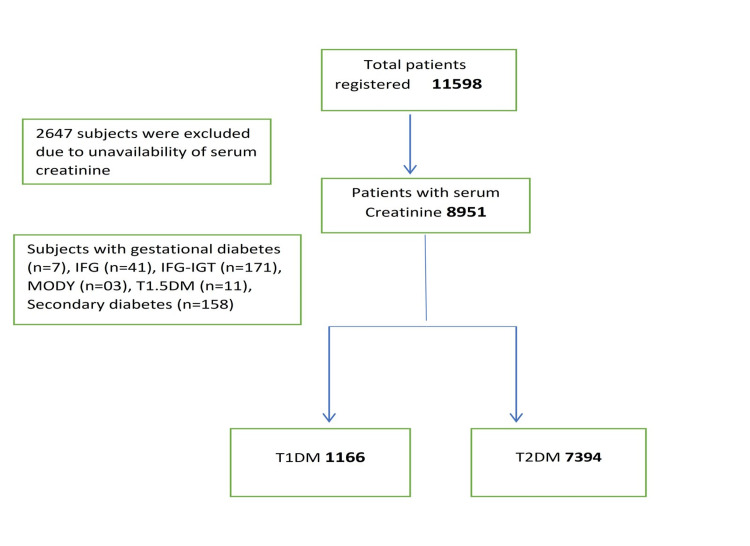
Flow sheet of total patients registered in DIAMOND database and process of selection of study cohort IFG: impaired fasting glucose; IFG-IGT: impaired fasting glucose-impaired glucose tolerance; MODY: maturity onset diabetes of young; T 1.5DM (type 1.5 diabetes mellitus); T1DM: type 1 diabetes mellitus; T2DM: type 2 diabetes mellitus (There were 6902 subjects with T2DM (as 492 subjects with unconfirmed T2DM diagnosis were excluded) and 1208 T1DM as 42 subjects with age 18 were included)

All patients 18 years or above with assigned diagnoses of either T1DM or T2DM with serum creatinine available in the database were included (Figure 1).

Exclusion Criteria

1. Patients with other types of diabetes except for T1DM and T2DM like MODY (Maturity onset diabetes of young), T1.5DM, gestational diabetes, latent autoimmune diabetes in adults (LADA), and diabetes due to secondary causes (pancreatitis, hemochromatosis, and drugs, etc.)

 2. Under 18 years of age

 3. Unavailability of serum creatinine

4. Patients with impaired fasting glucose (IFG) and impaired glucose tolerance (IGT)

Study design

An audit tool (Appendix 1) was developed and applied to the database in order to collect patients’ basic information including gender, age, type of diabetes, serum creatinine, urinary albumin excretion, and albumin creatinine ratio (ACR) against their medical record numbers (MRN). The latest entries which were available for each study subject in the database were used for analysis. Patients’ names and MRNs were removed to anonymize the data. Study subjects were divided into two cohorts (T1DM and T2DM) based on their diabetes. These cohorts were further subdivided into different stages of CKD depending upon their estimated glomerular filtration rate (eGFR) calculated by the modification of diet in renal disease (MDRD) formula [11]. 

As per guidelines eGFR <60ml/min or >60ml/min with positive ACR (evidence of structural kidney abnormality in normal or high eGFR) were labeled with CKD. CKD is defined as abnormalities of kidney structure or function present for > 3 months. Subjects with eGFR >60ml/min and negative or unavailable ACR values were not included in the results. Albumin creatinine ratio (ACR) on the spot urinary sample was used to define normal albumin excretion, moderately increased albumin excretion (microalbuminuria), and severely increased albumin excretion (macro albuminuria). ACR of > 2.5mg/mmol in males and > 3.5mg/mmol in females was taken as moderately increased albuminuria (microalbuminuria). Whereas, ACR > 20mg/mmol was defined as severely increased albuminuria (macro albuminuria) in both males and females [5]. The data was collected by using the DIAMOND database. A total of 7394 subjects in the T2DM cohort and 1166 in T1DM diabetes cohort were identified. Both cohorts were analyzed by applying an audit tool and were subdivided into different stages of CKD (CKD 1-CKD 5) on the basis of eGFR and ACR.

## Results

This study showed 42% of subjects with T2DM had CKD. Out of this cohort with CKD, 60% had diabetic kidney disease as manifested by albuminuria, and 40% with undetermined etiology. Similarly, there were a total of 18% of subjects with CKD in T1DM, 77.8% with diabetic kidney disease, and 22.2% with undetermined etiology.

CKD staging in the T2DM cohort

**Figure 2:**
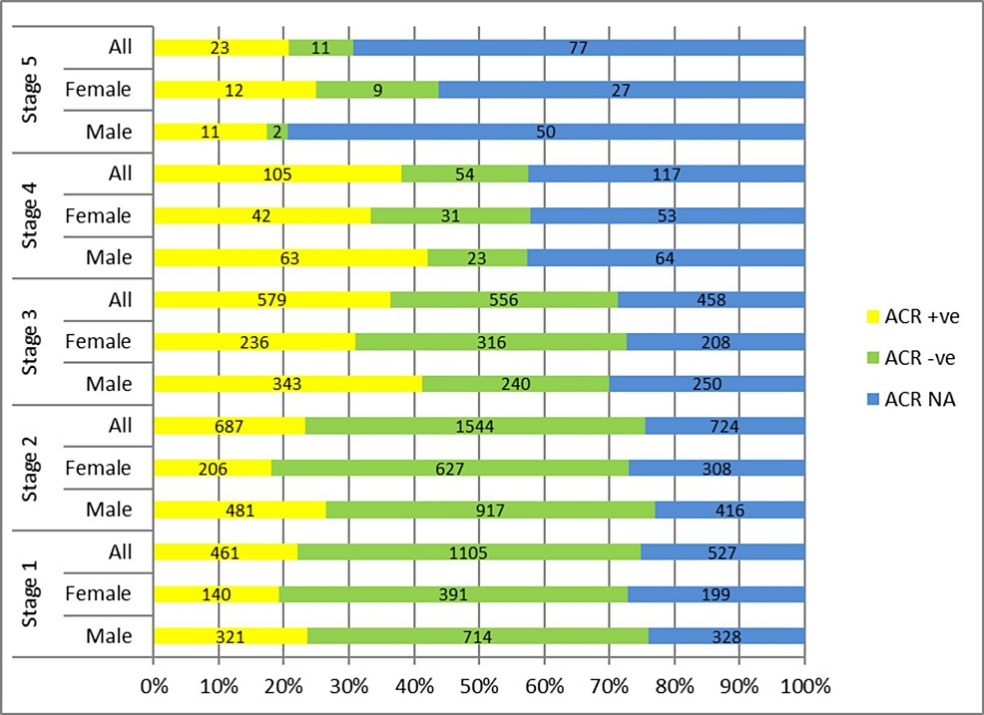
CKD STAGING BASED ON eGFR AND ALBUMINURIA IN T2DM COHORT CKD: Chronic kidney disease staging on vertical axis vs percentage of population on horizontal axis; ACR +ve: albumin Creatinine ratio positive; ACR-ve: albumin creatinine ratio negative; ACR NA: albumin Creatinine ratio not available

There were 7394 subjects in the T2DM cohort. Following KDIGO guidelines of CKD classification [12] subjects with eGFR > 60ml/min with no evidence of albuminuria (including both ACR negative and ACR NA) were excluded from CKD staging. So only ACR-positive subjects (determinant of structural kidney damage) in CKD 1 (n=461) and CKD 2 (n=687) with all subjects of CKD 3 (n=1593), 4 (n=276) and 5 (n=122) subgroups were included in determining the prevalence of CKD in T2DM cohort respectively (Appendix 2). Out of 7394 subjects with T2DM 3139 (42%) subjects were identified with CKD using these criteria (Appendix 1). The percentage of ACR-positive subjects increased with the worsening stage of the disease from CKD 1 to CKD 4 (from 22% to 38%) however this trend was not seen in CKD 5 subjects. Similarly, the percentage of ACR-negative subjects decreases with the worsening stage of disease from CKD 1 CKD 5. Taking ACR-positive status as a marker of diabetic kidney disease there were 1866 ACR-positive subjects out of 7395 (total subjects with T2DM). This shows that 25% of subjects have diabetic kidney disease in the T2DM cohort. There were 1903 subjects as shown in Appendix 2 with CKD 1-5 with no available ACR values in the database, constituting 37% of subjects with CKD (Figure 2).

CKD staging in the T1DM cohort

**Figure 3:**
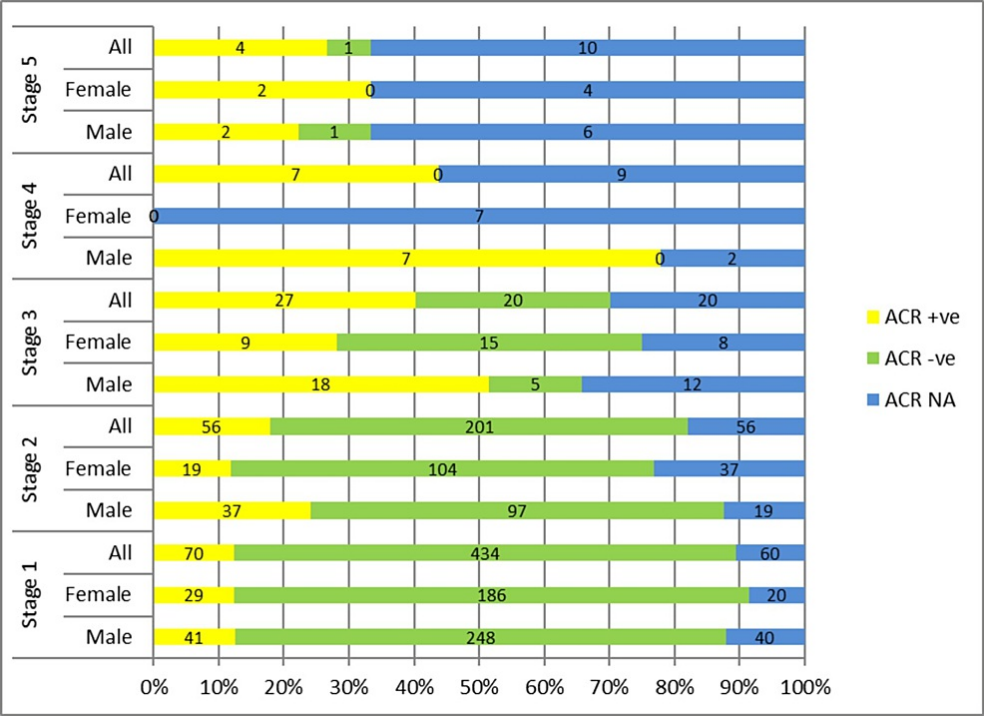
CKD STAGING BASED ON eGFR AND ALBUMINURIA IN T1DM COHORT CKD: Chronic kidney disease staging on vertical axis vs percentage of population on horizontal axis; ACR +ve: albumin Creatinine ratio positive; ACR-ve: albumin creatinine ratio negative; ACR NA: albumin Creatinine ratio not available

There were 1166 subjects with T1DM of aged 18 years or above. Similarly, subdivisions in terms of albuminuria (ACR Positive) were as follows: CKD 1 (n=70), CKD 2 (n=56), CKD 3 (n=27), CKD 4 (n=7), and CKD 5 (n=4). Following KDIGO guidelines of CKD classification [4] subjects with eGFR >60ml/min with no evidence of albuminuria (including both ACR negative and ACR NA) were excluded from CKD staging. So only ACR-positive subjects (determinant of structural kidney damage) in CKD 1 ( n=70) and CKD 2 (n=56) with all subjects of CKD 3 (n=27), 4 (n=7) and 5 (n=4) subgroups were included in determining the prevalence of CKD in T1DM cohort, respectively. Out of 1166 study subjects with T1DM, 209 (18%) subjects were identified with CKD using KDIGO criteria (Appendix 3). The percentage of ACR-positive subjects increased with the worsening stage of the disease from CKD 1 to CKD 4 (from 12% to 44%) however this trend was not seen in CKD 5 subjects. Similarly, the percentage of ACR-ve subjects also increased from CKD 1 to CKD 4. Taking ACR-positive status as a marker of diabetic kidney disease there were 164 ACR-positive subjects out of 1166 (total subjects with T1DM). It shows 14% of subjects have diabetic kidney disease in the T1DM cohort. There were 155 subjects in Appendix 2 with CKD 1-5 with no available ACR values in the database, constituting 13% of subjects with CKD (Figure 3).

Prevalence of CKD in T2DM and T1DM cohorts

The overall prevalence of CKD in T2DM and T1DM cohorts which were identified in 42% and 18% of subjects, respectively.

Relationship of Age with Type of Diabetes, CKD and Albuminuria

**Figure 4:**
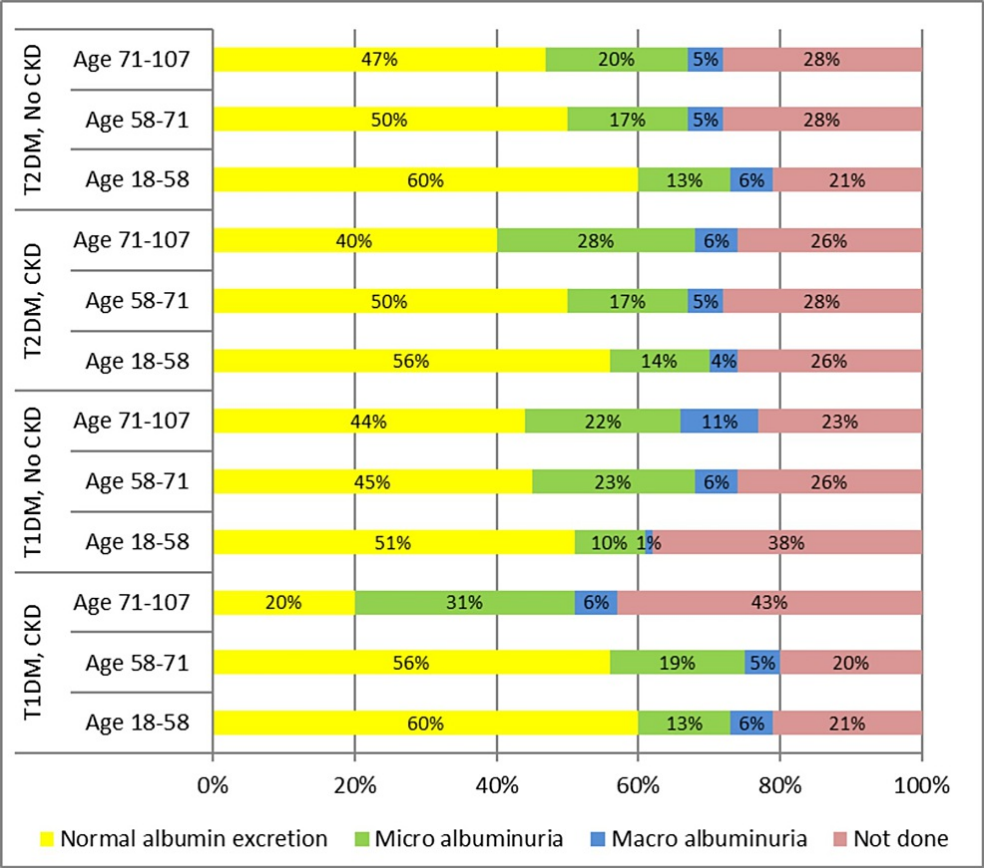
Relationship of age with type of diabetes, CKD and albuminuria CKD: Chronic kidney disease staging on vertical axis vs percentage of population on horizontal axis; ACR +ve: albumin Creatinine ratio positive; ACR-ve: albumin creatinine ratio negative; ACR NA: albumin Creatinine ratio not available

In order to evaluate the relationship of age with type of diabetes, CKD staging and albuminuria (both micro and macro albuminuria) subjects from the DIAMOND database with both type2 DM and type1 DM (n=8110) were divided into the following three age bands 18-58 years, 58-71 years and 71-107 years in order to see any change in urinary albumin excretion with aging. CKD was defined as eGFR < 60ml/min [13], micro- and macro albuminuria were respectively defined as ACR > 2.5(M), > 3.5(F) and > 20mg/mmol [14]. The last available serum creatinine and urine albumin/creatinine ratio (UACR) in patients with T1DM or T2DM diabetes recorded on the DIAMOND database were used for analysis. Both T1DM and T2DM subjects were combined in a single cohort with subdivision into three age categories from 18 years (adult age) to 107 years (maximum registered age in the database). The division of the total population into three age categories of equal numbers helped in evaluating the relationship between age with type of diabetes,

CKD and albuminuria (Appendix 4). The prevalence of CKD and albuminuria was related to age in both T1DM and T2DM populations. Albuminuria showed a linear relationship with age in subjects with no known CKD independent of type and duration of diabetes. CKD was common and increased with age in the study cohort. Rates of microalbuminuria also increased with age and were similar in patients with and without CKD. Macroalbuminuria was relatively uncommon (Figure 4).

## Discussion

This study set out to identify the prevalence of CKD and albuminuria in T1DM and T2DM subjects attending tertiary diabetic units in the Republic of Ireland. It also looked into the relationship of age with type of diabetes, CKD, and albuminuria in both CKD and non-CKD subjects in the study cohort. CKD was determined by eGFR calculated by a modified MDRD equation based on the last available serum creatinine and albuminuria by spot urinary albumin to creatinine ratio (ACR). We found CKD was common in both groups with a higher prevalence of albuminuria in T1DM.

UKPDS (United Kingdom Prospective Diabetes Study), one of the largest prospective studies in T2DM subjects with nearly 5100 subjects enrolled from the time of diagnosis of T2DM reported a prevalence of microalbuminuria of 25%, macro albuminuria of 8%, and raised serum creatinine (defined as ≥ 175 micromole/l) or requirement of renal replacement therapy of 0.8% at 10 years following diagnosis of T2DM [10]. UKPDS also determined yearly progression of diabetic nephropathy from diagnosis to microalbuminuria, from microalbuminuria to macro albuminuria, and from macro albuminuria to elevated serum creatinine or renal replacement therapy was 2.0, 2.8, and 2.3% respectively.

Although albuminuria (ACR+ve status) is a hallmark of DKD, there are studies that have shown that decline in eGFR <60ml/min in the absence of other causes of CKD was seen in T2DM with no evident micro/macro albuminuria [15-17]. The National Evaluation of the Frequency of Renal Impairment co-existing with NIDDM (NEFRON) found that 55% of subjects with CKD had normal albumin excretion in T2DM [18]. In Third National Health and Nutrition Examination Survey (NHANES III), 1197 subjects with T2DM and low eGFR (<60ml/min) were present in 30% of subjects in the absence of micro- or macro albuminuria and retinopathy [19]. These studies showed that the absence of albuminuria in T2DM subjects did not rule out DM as a cause of CKD. Therefore in light of their findings, there is a possibility that a reasonable percentage of subjects with non-albuminuric CKD in our T2DM cohort might have diabetes as a likely etiology.

This study clearly shows that the percentage of albuminuria (ACR+ve status) increases with the worsening of the disease state from CKD 1-CKD 4. This trend however was not seen in CKD 5 subjects which may be due to the fact that as ACR is a screening test for albuminuria and subjects with CKD 5 have advanced kidney disease, they may have had more advanced diagnostic work-up like 24-hour urine collection for proteins, renal imaging and prerenal replacement therapy/transplant investigations at that stage. The “ACR NA” status of 63% in CKD 5 subjects supports this assumption. 

The prevalence of CKD in T1DM cohort was found to be 18% out of 1,166 T1DM subjects registered in the DIAMOND database. As albuminuria is the pathognomonic marker of diabetic kidney disease, there were 14% of subjects were identified with albuminuria (ACR +ve status) and labeled with DKD. CKD in T1DM subjects was largely related to DM. This finding is different from the T2DM cohort with only a 25% prevalence of DKD based on albuminuria (ACR+ve status) but with an overall prevalence of CKD of 42%. The possible explanation for the higher prevalence of non-albuminuric CKD in the T2DM cohort could be due to the older age of the T2DM cohort (Appendix 1), the presence of hypertension, dyslipidemia, peripheral vascular disease, and greater exposure to other risk factors for CKD. Studies have shown that aging is associated with a decrease in renal mass, blood flow, and creatinine clearance [20-21].

There were 14% of subjects with CKD in T1DM that had positive urine albumin excretion. Studies have shown that microalbuminuria begins five to 15 years after the onset of diabetes in T1DM subjects and it increases over time [22]. In a systematic review of 9 longitudinal studies with 7938 T1DM subjects, the prevalence of microalbuminuria was 28% at 15 years after the onset of diabetes [21]. In another study on T1DM, the prevalence of microalbuminuria was 52% at 30 years [3]. There are multiple studies in T1DM subjects showing that improved glycaemic control and the addition of ACEI (angiotensin-converting enzyme inhibitors) or ARB (angiotensin receptor blockers) for blood pressure are associated with delay in progression or even regression in DKD [22,23]. Albuminuria is an independent risk factor for cardiovascular disease and a marker of endothelial dysfunction [24]. The higher prevalence of albuminuria in T1DM cohort in comparison with T2DM cohort in reference to the overall CKD population in both groups adds an additional risk factor to a former group for cardiovascular disease. In a systematic review with 7938 subjects, the relative risk of all-cause mortality and cardiovascular disease was 1.8 (95% CI 1.5-2.1) in subjects with microalbuminuria compared to normoalbuminuria [5]. The risk of other microvascular complications, such as retinopathy and neuropathy is increased in subjects with microalbuminuria [25]. Early detection and management of microalbuminuria are important to prevent its progression to worsening kidney disease. Studies have shown that better glycaemic and blood pressure control decreases CKD progression and cardiovascular disease [26].

Strengths and limitations of this study

This is the first study that has evaluated the prevalence of CKD in a DM population in the Republic of Ireland. In addition, this has a large sample (n=7394 T2DM and n=1166 T1DM subjects) with the availability of serum creatinine and eGFR in all subjects and ACR values in 81% of the population with gender distribution in each stage of CKD. 

eGFR was estimated by a modified MDRD equation and albuminuria by ACR. ACR has shown high sensitivity and specificity in defining micro and macro albuminuria [4]. ACR status was evaluated in both genders with numbers and percentages of each category. KDIGO guidelines were validated in the evaluation and staging of CKD with combined criteria of eGFR (as a marker of kidney function) and albuminuria (as a marker of kidney structure). Data were also analyzed in terms of three age bands of equal numbers separately to evaluate the relationship of age with type of diabetes, CKD, and albuminuria in a combined cohort of T1DM and T2DM population.

There are several limitations to the study. KDIGO defines CKD as ‘’abnormalities of kidney structure or function, present for > 3 months, with implications for health ’’ [4], so two serum creatinine values and ACR values are important in staging CKD. In this study CKD staging and albuminuria are based on the last available values in the DIAMOND database which is a potential limitation of this study. Secondly, information on the duration of diabetes in both T1DM and T2DM cohorts was not available; however, it could be indirectly estimated from the date of the first registration of each subject into the database. Thirdly ethnicity is not recorded in the DIAMOND database so the option of ‘’other’’ was used in calculating eGFR. According to the national census population 2016, there are 91.7% white population and the rest are of other ethnicities including non-Chinese Asians, mixed and Chinese [26]. fourthly a proportion of each of the T1DM and T2DM cohorts did not have their albuminuria status: Albumin Creatinine Ratio Not Available (ACR NA) registered in the database. Albuminuria is one of the earliest signs of diabetic kidney disease with normal eGFR so the non-availability of ACR in roughly 20-30% of the population may affect the actual prevalence of diabetic kidney disease in the study cohort. Although there was a lack of data in terms of albuminuria (ACR status or 24-hour urinary proteins) in Stage 5 CKD subjects from both T1DM and T2DM cohorts, previously explained these subjects had advanced investigations including 24-hour urinary protein, US kidneys, and renal replacement therapy or transplant work up rather than ACR which is a screening test for albuminuria. However, since this is the first study of its kind to be undertaken in an Irish diabetic population the results will help to provide some baseline data on the prevalence of CKD in these patients, which is important for healthcare planning. 

In summary, this study, involving large numbers of T1DM and T2DM patients showed that CKD was common in both cohorts. The prevalence of CKD and albuminuria increased with age. These results suggest that a significant proportion of T1DM and T2DM patients have CKD. Since CKD is independently associated with cardiovascular disease and death, its early detection and management significantly reduce morbidity and mortality [27]. Therefore, T1DM and T2DM patients should be proactively monitored for both microvascular and macrovascular complications of diabetes.

## Conclusions

CKD was noted to be common in both cohorts with higher prevalence in T2DM population. The prevalence of albuminuria was more common in T1DM population. Both CKD and albuminuria were related to age in both T1DM and T2DM populations.

## Appendices

Appendix 1 

**Table 1:** Audit Tool MRN: medical record number; serum creatinine measured in micromole/litre; eGFR estimated glomerular filtration rate measured in mL/min per 1.73 m2; CKD chronic kidney disease; ACR albumin creatinine ratio measured in mg/mmol; A1 normoalbuminuria A2 moderately increased albuminuria (micro albuminuria) A3 severely increased albuminuria (macroalbuminuria)

Name	MRN	gender	age	Type of diabetes	Serum Creatinine	eGFR /CKD Staging	ACR	A1/A2/A3

Appendix 2 

**Table 2:** CKD staging based on eGFR and albuminuria in T2DM cohort

ACR status	Stage 1 N=2093	Stage 2 N= 2955	Stage 3 N=1593	Stage 4 N=276	Stage 5 N= 122
ACR positive	461(22%)	687(23%)	579(36%)	105(38%)	34(28%)
ACR negative	1105(53%)	1544(52%)	796(35%)	54(20%)	9(9%)
ACR NA	527(25%)	724(25%)	458(29%)	117(42%)	77(63%)

Appendix 3 

**Table 3:** CKD staging based on eGFR and albuminuria in T1DM cohort

ACR status	Stage 1 N=564	Stage 2 N= 313	Stage 3 N=67	Stage 4 N=16	Stage 5 N= 15
ACR positive	70(12%)	56(18%)	27(40%)	7(44%)	4(27%)
ACR negative	434(77%)	201(64%)	20(30%)	0(0%)	1(6%)
ACR NA	60(11%)	56(18%)	20(30%)	9(56%)	10(66%)

Appendix 4 

**Table 4:** Relationship of age with type of diabetes, CKD and albuminuria NA-normal albumin excretion; MA-micro albuminuria; A-macro albuminuria; ND-not done ** There were 6902 subjects with T2DM (as 492 subjects with unconfirmed T2DM diagnosis were excluded) and 1208 T1DM as 42 subjects with age 18 were included.

	T1DM, CKD	T1DM, No CKD	T2DM, CKD	T2DM, No CKD
	Number/ % (%NA/MA/A/ND)	Number/ % (% NA/MA/A/ND)	Number/ % (%NA/MA/A/ND)	Number/ % (%NA/MA/ND)
Age 18-58 (n= 2702)	271/ 27% (60/ 13/ 6/ 21)	732/ 73% (51/ 10/ 1/ 38)	721/ 42% (56/ 14/ 4/ 26)	978/ 58% (60/ 13/ 6/ 21)
Age 58-71 (n= 2704)	106/ 75% (56/ 19/ 5/ 20)	36/ 25% (45/ 23/ 6/ 26)	1736/ 67% (50/ 17/ 5/ 28)	826/ 33% (50/ 17/ 5/ 28)
Age 71-107 (n= 2704)	54/ 85% (20/ 31/ 6/ 43)	9/ 15% (44/ 22/ 11/ 23)	2244/ 84% (40/ 28/ 6/ 26)	397/ 16% (47/ 20/ 5/ 28)
